# Reptiles as Promising Sources of Medicinal Natural Products for Cancer Therapeutic Drugs

**DOI:** 10.3390/pharmaceutics14040874

**Published:** 2022-04-16

**Authors:** Soon Yong Park, Hyeongrok Choi, Jin Woong Chung

**Affiliations:** 1 Research Center, Dongnam Institute of Radiological & Medical Sciences, Busan 460333, Korea; sypark@dirams.re.kr; 2 Department of Biological Science, College of Natural Sciences, Dong-A University, Busan 49315, Korea; gwangdoli@naver.com

**Keywords:** natural drugs, cancer therapy, reptile-derived components, extracts, crude peptides, sera, venom

## Abstract

Natural products have historically played an important role as a source of therapeutic drugs for various diseases, and the development of medicinal natural products is still a field with high potential. Although diverse drugs have been developed for incurable diseases for several decades, discovering safe and efficient anticancer drugs remains a formidable challenge. Reptiles, as one source of Asian traditional medicines, are known to possess anticancer properties and have been used for a long time without a clarified scientific background. Recently, it has been reported that extracts, crude peptides, sera, and venom isolated from reptiles could effectively inhibit the survival and proliferation of various cancer cells. In this article, we summarize recent studies applying ingredients derived from reptiles in cancer therapy and discuss the difficulties and prospective development of natural product research.

## 1. Introduction

Cancer, an intricate genetic disease triggered by mutations that create expression, functional, and/or structural abnormalities in major genes, remains an unconquered disease worldwide [1]. Cancer can promote recurrence and metastasis via uncontrolled growth, high motility, and various survival defense mechanisms induced through complex genetic modifications [2,3,4]. Over the last several decades, highly efficacious cancer therapeutic methods have been developed. Currently, synthetic chemical compounds are commonly used for effective cancer treatment, but they are accompanied by serious side effects, including vomiting, loss of hair and body weight, and functional impairment of organs. Molecular targeted therapy is a novel approach in which molecules specifically bind to membrane proteins, such as epidermal growth factor receptor (EGFR), vascular endothelial growth factor receptor (VEGFR), platelet-derived growth factor receptor (PDGFR), anaplastic lymphoma kinase (ALK), and human epidermal growth factor receptor-2/-3 (HER-2/-3). Alternatively, the molecules inhibit specific signal transducers, including proto-oncogene tyrosine kinase (SRC), proto-oncogene serine/threonine kinase (RAF), phosphoinositide 3-kinase (PI3K), mammalian target of rapamycin (mTOR), mitogen-activated protein kinase 1/2 (MEK1/2), mitogen-activated protein kinase (MAPK), the cyclin D1/cyclin-dependent kinase 4 (CDK4), and cyclin-dependent kinase 6 (CDK6) [5,6,7,8]. Although molecular targeted therapies have fewer side effects than chemical compounds alone, it is difficult to establish a standard therapeutic method because their efficacy is different depending on the tumor type [9,10,11]. Additionally, it might be difficult to find a proper therapeutic target which is at a significantly high level or only expressed in a specific tumor. Cancer immunotherapy is a recently developed technology that suppresses the immune-evasion mechanism of cancer cells and improves the activity of immune cells [12,13,14,15]. For example, immunotherapy can suppress tumor growth by activating T cells via the inhibition of programmed cell death protein-1/programmed cell death-ligand 1 (PD-1/PD-L1). This binding of cancer cells and T cells is an important immune checkpoint mechanism [16,17,18]. Additionally, chimeric antigen receptor T (CAR-T)-cell therapy is an innovative personalized cancer treatment. T cells that have been isolated from the blood of patients or donors are genetically engineered to enhance their expression of specific receptors and cytotoxicity against cancer cells [19,20,21]. This therapy is a cell-based technology without any cytotoxicity to normal organs. It can be customized according to the type of tumor and the patient’s condition. Despite these advantages, the CAR-T-cell technique is only effective in some blood cancers, such as leukemia, and it has even resulted in the acceleration of tumor progression. Therefore, it is necessary to develop materials or medical technologies that are capable of effectively targeting tumors alone and not normal tissues and organs.

Throughout human history, natural products have been used in various forms from a time when there was no clear scientific verification for their use to the present. Based on the knowledge acquired through experience, natural products have been used for the prevention and treatment of diverse disorders, such as inflammation, hyperpyrexia and infection [22,23,24,25]. With advances in science and technology, it has become possible to select, isolate, and purify key factors possessing medical therapeutic effects. Because natural products are derived from microbes, plants, marine organisms and animals, they have superior physiological stability and safety. Based on these strengths, a number of pharmaceutical companies and research institutes have made various attempts to discover powerful anticancer factors from natural products [26,27]. Many types of active anticancer components derived from plants have already been developed as therapeutic drugs and are currently being used to cure patients. Additionally, marine natural product research, including research on microorganisms, phytoplankton, algae, sponges and fish, has progressed, and the functional roles of bioactive compounds have been clearly verified. Although fewer anticancer molecules have been derived from animals than from plants and marine organisms [28,29,30,31,32], animal-derived components have some benefits, such as efficient biocompatibility, bioactivity, stability and safety to human tissues [33]. Reports have proven the bioactivity of peptides that have been isolated from goat spleens or livers and bovine meat [34,35]. Lactoferrin, which is known to be contained in large amounts in colostrum, has been proven to be a multifunctional glycoprotein that inhibits senescence, inflammation and tumors [36]. Surprisingly, diverse anticancer peptides have been identified from the skin secretions of amphibians. Reptiles have also been used in Asian traditional medicine along with amphibians [37,38,39]. Reptile-derived bioactive components have been isolated through different extraction processes, and their therapeutic effects on cancer have been reported in the past two decades. Anticancer research using reptile-derived components has abundant potential as well as a high value because it is still in the early development stage [40,41].

Here, we discuss the development of cancer therapeutic drugs that are based on bioactive natural products and derived from sources including plants, marine organisms and animals. Specifically, we focus on recent research that has uncovered anticancer components derived from reptiles that may help to overcome challenges in cancer therapy.

## 2. Natural Sources of Bioactive Cancer Therapeutic Components

### 2.1. Plants

Since ancient times, terrestrial plants have been a very important source of medicines used for preventing or treating various diseases. Several anticancer drugs currently in use are derived from terrestrial plants (Table 1). Vincristine and vinblastine, typical anticancer drugs containing components derived from plants, are vinca alkaloids derived from the leaves of *Catharanthus roseus* (also known as *Vinca rosea* or the Madagascar periwinkle plant). Vincristine and vinblastine are effective against some types of cancer, such as acute leukemia, Hodgkin lymphoma, lymphosarcoma, testicular cancer and lung cancer [42,43]. They inhibit the growth of cancer cells by inhibiting tubulin function during cell division. Paclitaxel was isolated from *Taxus brevifolia*, the pacific yew. Paclitaxel is an antiproliferative drug that promotes microtubule assembly and stability, and it has been reported to be effective in the treatment of ovarian, cervical, breast, lung and pancreatic cancer [44]. In addition, numerous plant-derived anticancer components, such as resveratrol, curcumin, epigallocatechin gallate, quercetin, rutin and ginsenoside, have been discovered and developed as drugs [45,46,47,48,49,50,51,52,53]. Many types of terrestrial plant-derived anticancer components have also been discovered and developed as effective drugs. Therefore, in the future, research on the development of drugs derived from plant components might have great potential to improve efficiency in applied studies.

### 2.2. Marine Organisms

Marine compounds possess unlimited potential for drug discovery because there are likely to be many unknown organisms and untapped areas of research. Marine research for drug development has been underway since the 1980s. Various anticancer peptides have been isolated from diverse marine organisms such as algae, sponges and fish (Table 2). Lurbinectedin is an alkaloid isolated from *Ecteinascidia turbinata* that might promote cancer cell death by inhibiting gene transcription [29]. Tisotumab vedotin, known by the brand name Tivdak, is an antibody–drug conjugate (ADC) used to treat cervical cancer. Tisotumab vedotin that has been isolated from *Dolabella auricularia* might induce apoptosis through cell cycle arrest by binding to the tissue factor (CD142) on the cancer cell surface and releasing monomethyl auristatin E (MMAE) in the cell [29]. Additionally, many types of alkaloids, peptides and ADC anticancer drugs are being continuously developed. According to recent reports, many marine natural products isolated from fish, seaweed, fungi, mangroves, microalgae, cone snails, sea hares and mollusks can inhibit cancer progression both in vivo and in vitro through cell lysis, necrosis and apoptosis [54,55,56,57,58,59]. Marine bioactive components might attenuate several types of cancer cells, such as breast, lung, bladder, prostate, melanoma and leukemia cells [60,61,62]. It is expected that marine natural products will continue to have high potential because there are still numerous unexplored marine organisms.

## 3. Cancer Therapeutic Research Using Reptile-Derived Components

Drugs derived from reptiles have long been widely used as a source of nourishment, nutritional tonics and disease treatment in Asia. In general, powders and other derivatives can be made from dried reptiles after the removal of the intestines, or extracts can be created by soaking the whole reptile in alcohol. Recently, the inflammatory and tumor-suppressive effects of components derived from reptiles have been experimentally proven, and anticancer research is being conducted with various formulations generated with different extraction methods. Typically, cancer therapeutic studies are performed using extracts, crude peptides, sera and/or venom isolated from reptiles through several extraction methods (Table 3).

### 3.1. Extracts

Reptile-derived extracts, which are very similar to traditionally derived agents, have been proven to have excellent tumor-suppressive effects. According to the reported studies, aqueous extracts of *Gekko swinhonis* inhibited the proliferation and differentiation of human hepatic cancer cell lines such as L-02 and Bel-7402 [72]. Additionally, aqueous extracts of *Gekko subpalmatus Gunther* exhibited antitumor effects in vivo and inhibited the growth of the Bel-7402 cell line [73]. Moreover, they decreased the expression of alpha fetoprotein (AFP), which is a malignant phenotype marker of liver tumors. Extracts of *Gekko japonicus* showed antitumor effects against the human esophageal carcinoma EC9703 and mouse sarcoma S180 cell lines in experiments in vitro and in vivo [74]. Surprisingly, extracts derived from *Cyrtopodion scabrum* using ethanol and distilled water showed selective anticancer effects against the human breast cancer cell line MCF7 and human colon cancer cell line SW-742 through the inhibition of growth and migration, and they had no effect on normal mesenchymal stem cells [75]. In addition, the aqueous protein extracts of *Eublepharis macularius* induced apoptosis only in human cancer cell lines, such as bladder cancer 5637 and cervical cancer HeLa cells, via the inhibition of the PI3K/Akt signaling pathway, and without any effect on normal cells, including C2C12, NIH3T3, MEF, HEK293, Hs27 and NuFF cells [76,77]. Jeong et al. and Kim et al., proved the anticancer effects of proteins or peptides through several experiments using heat-inactivated extracts. Furthermore, these extracts suppressed the proliferation and survival of cancer cells through G2/M cell cycle arrest and the caspase-dependent apoptosis pathway via the inhibition of PI3K/Akt signaling in the human non-small-cell lung cancer cell line A549 and the mouse lung cancer cell line Lewis lung carcinoma (LLC). In particular, the intravenous injection of these protein extracts showed pharmaceutical potential by reducing tumor volume via the suppression of Akt phosphorylation in vivo. According to this paper, Lee et al. performed the fractionation and isolation of reptile extracts, and several candidates were identified as major anticancer components. Additionally, they identified the various proteins contained in the extracts by performing proteomics analyses. This revealed the interaction network, the biological process that categorizes differentially expressed proteins and the involved cellular-signaling pathways of all identified proteins contained in the extracts [78]. Finally, these studies explicitly demonstrated that the aqueous protein extracts of *Eublepharis macularius* might possess therapeutic effects against many types of human cancer cells via in vitro and in vivo experiments.

Crocodile choline, a bioactive component derived from *Crocodylus siamensis*, promoted cell death in gastric cancer cell lines, including the BGC823, MGC803, SGC7901 and MKN28 cell lines, without side effects on the normal gastric cell line GESI [79]. This agent also induced apoptosis through G2/M cell cycle arrest in the BGC823 cell line. In experiments conducted in vivo, the intragastric (i.g.) administration of crocodile choline not only suppressed tumor growth but also had no striking effects on other internal organs. Additionally, the lysates of several organs of *Crocodylus palustris*, such as the heart, lungs, intestine and brain, showed a high cytotoxicity against the human prostate cancer cell line PC3 [80]. In addition, the aqueous extracts of white blood cells derived from *Crocodylus siamensis* induced apoptosis in the human cervical cancer cell line HeLa through the mitochondria/caspase-3/caspase-9-mediated intrinsic pathway and effectively inhibited the proliferation, migration and invasion of cancer cells [81,82]. Furthermore, the extracts promoted apoptosis in the human cancer cell lines LU1, LNCaP, PC3, MCF and CaCo2 through G2/M phase cell cycle arrest [83]. These studies suggest that various extracts derived from crocodile might be useful materials in the development of pharmaceuticals.

Aqueous extracts of turtle shell derived from *Chinemys reevesii*, *Cuora aurocapitata* or *Trachemys scripta* promoted cell death in the human leukemia cell line HL60 and the human liver cancer cell line HepG2 [84]. Although research is still in the early stages, these studies suggest that extracts of reptiles could be effective drug candidates for cancer therapy.

### 3.2. Crude Peptides

Peptide mixtures or crude peptides isolated from reptiles may play an important role in drug development and might provide promising options for cancer therapy. Protein and functional peptides are safe drugs that have an excellent cell permeability and minimal potential to elicit an immune-rejection response. Therefore, peptides derived from reptiles could be key drug candidates. A sulfated polysaccharide–protein complex can be extracted from the dried whole body of *Gekko swinhonis Guenther* using polar solvents. Several features of the protein complex were analyzed through high-performance liquid chromatography, gas chromatography–mass spectrometry, gas chromatography and NMR spectroscopy. Remarkably, the complex inhibited the proliferation and migration abilities of the human hepatocellular carcinoma cell line SMMC-7721 [85]. Additionally, the crude peptides isolated from alcohol extracts of dried whole *Gekko japonicus* suppressed the proliferation of the human liver carcinoma cell line HepG2 and promoted apoptosis through Bcl-2/Bax pathway regulation [86]. These crude peptides significantly reduced the tumor weight as well as the expression of VEGF in xenograft tumors derived from the mouse liver carcinoma cell line H22. Similarly, a polypeptide mixture extracted from *Gekko japonicus* powder effectively inhibited the proliferation and induced the apoptosis of the human liver carcinoma cell line HepG2 [87]. Interestingly, differentially expressed genes in HepG2 cells treated with the polypeptide mixture were analyzed through RNA-Seq, gene ontology and protein–protein interaction analyses. Additional experiments demonstrated that several genes involved in the apoptosis induced by the peptide mixture promote reactive oxygen species (ROS)-related processes and the unfolded protein response (UPR). These studies show that if the identity of reptile-derived peptides is determined, valuable drugs based on these targets and related signaling pathways could be developed.

### 3.3. Sera and Bile

Anticancer studies based on body fluids rather than whole-body-derived components of reptiles are rare because a relatively fresher sample is needed. Nevertheless, studies have verified the anticancer properties of reptile serum and bile. Serum isolated from *Varanus salvator* blood showed cytotoxicity against HeLa cells without affecting the normal human keratinocyte cell line HaCaT [88]. The sera of *Malayopython reticulatus* and *Cuora amboinensis kamaroma* also exerted anticancer effects against the human cancer cell lines HeLa, PC3 and MCF7 [88]. This study identified several compounds, including purine, alpha-naphthylthiourea (ANTU), 6E, 9E-octadecadienoic acid, allo-inositol and uric acid, that possess various bioactivities from the sera of these reptiles using LC–MS/MS analysis.

Animal bile has been used as a natural drug to strengthen stamina and immunity for thousands of years. Bile contains many bile acids, such as cholic acid and deoxycholic acid, that play a role in physiological functions. Bile juice squeezed from the gallbladders of *Crocodylus siamensis* promoted apoptosis in the human non-small-cell lung cancer cell line NCI-H1299 through the mitochondria/caspase-3/caspase-9-mediated intrinsic pathway, and effectively suppressed tumorigenesis and growth in athymic nude mice [89]. These findings could expand the pool of natural drug development for cancer therapy in the near future.

### 3.4. Venom

Effective therapeutic factors that have been diluted or purified from venom have been used to treat diseases. Bee venom, for example, is composed of approximately 40 factors, and among these, melittin and apamin may enhance the immune system through the promotion of hormone metabolism, blood circulation and immune cell activation. This occurs by stimulating the anterior pituitary or adrenal cortex [109,110]. Botulinum toxin derived from *Clostridium botulinum* is a neurotoxic protein that blocks the release of acetylcholine from the axon terminal of neurons. Botulinum toxin types A and B are applied in the medical field to treat the hyperactive responses of muscles and neurons [111,112,113]. Research on reptile-derived venom has also been conducted, and several effects on cancer cells have been demonstrated. Lectin purified from *Macrovipera lebetina*, BnSP-6 and Lys-49 phospholipase A2 (PLA2) purified from *Bothrops pauloensis*, NN-3 purified from *Naja naja oxiana*, and chlorotoxin purified from *Leiurus quinquestriatus* were analyzed to determine their structure, amino acid sequence and anticancer effect against MDA-MB-231 cells [90,91,92,93]. Additionally, macrovipecetin, a C-type lectin from *Macrovipera lebetina*, suppressed the migration, invasion and proliferation of the human melanoma cell line SK-MEL-28 [94]. Crotoxin, a toxin derived from *Crotalus durissus terrificus* and cytotoxin 2 derived from *Naja naja oxiana* decreased the viability of MCF7aro cells via the inhibition of the extracellular signal-regulated kinase (ERK) pathway, inhibited cell migration by inducing the apoptosis of MCF7 cells, and showed an anticancer effect on lung cancer cells in A549 cells through the activation of the caspase-3 and p38 pathways [95,96,97]. Daboialectin, a C-type lectin derived from *Daboia russelii*, effectively inhibited cell motility by promoting the cytoskeletal damage and apoptosis of A549 cells [98]. Additionally, cytotoxin 1 (a polypeptide consisting of 60 amino acids from *Naja atra*) caused the necroptosis of the leukemia cell lines HL-60 and KG1a [99]. Disintegrin isolated from *Echis multisquamatus* exerted antiproliferative action against HeLa cells [100]. The venom extracts of *Vipera latifii* or *Naja hage* showed anticancer effects against the hepatocellular carcinoma cell lines HepG2 and Huh7.5 [101,102]. Additionally, the venom extracts of *Walterinnesia aegyptia* induced apoptosis through the activation of the caspase-3 pathway in human breast cancer cell lines MDA-MB-231 and MCF7 [103]. The novel recombinant protein cytotoxin 2 derived from *Naja naja oxiana* inhibited the transforming growth factor/suppressor of mothers against the decapentaplegic (TGFβ/SMAD) signaling pathway, and the expression of matrix metalloproteinase 3 (MMP3) in the human melanoma cell line SK-MEL-03, without side effects on the normal fibroblast cell line HFF-2 [104]. Jararhagin toxin from *Bothrops jararaca* reduced proliferative ability through the activation of the caspase-3 pathway in the murine melanoma cell line B16F10 [105]. L-Amino acid oxidase isolated from *Cerastes vipera* showed powerful cytotoxicity against MCF7, HepG2, A549, and PC3 cells and the human colon cancer cell line HCT116, compared with doxorubicin [106,107]. The activated crude venom of *Cerastes cerastes* by irradiation (Co-60 gamma rays) induced apoptosis via G2/M phase cell cycle arrest in the human non-small-cell lung cancer cell line A549 and the human prostate cancer cell line PC3 [108]. These studies demonstrated that isolation, purification and functional peptide analysis can be used to develop diverse venom-derived products as effective cancer therapeutic drugs.

## 4. Conclusions and Perspectives Regarding Reptile-Derived Products as Natural Pharmaceutical Materials

**Figure 1:**
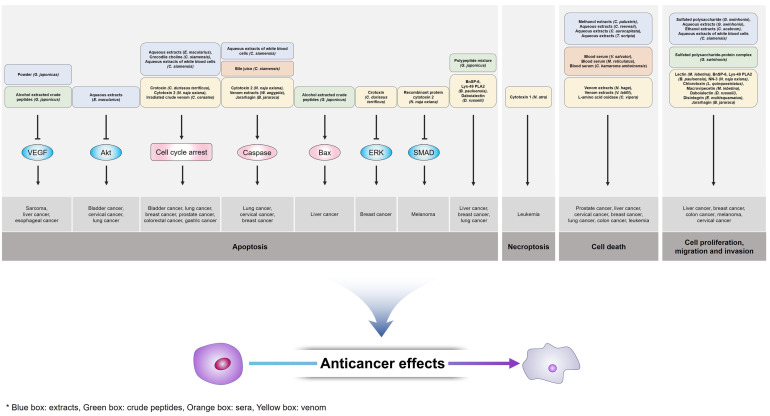
Schematic image of anticancer effect mechanisms of reptile-derived components.

Therapeutic bioactive components derived from various reptiles are valuable natural sources with the potential to overcome the shortcomings of current cancer therapies (Figure 1). In the past, reptile components have been used in the form of extracts, powders, pills and liquors. Additionally, toxic ingredients in venom have been diluted or weakened for use. In Asian medicine, reptile-derived components are known to have pharmaceutical effects against several disorders, such as inflammation, nephrolithiasis, eczema and tumors. In recent years, advanced analysis techniques have made it possible to isolate components derived from reptiles and identify their pharmacological effects, structures, functions, and mechanisms. Interestingly, studies have structurally minimized fragments of crotamine and crotalicidin isolated from *Crotalus durissus terrificus* venom for application in medicinal technology [114]. However, the development of reptile-derived anticancer components is still in the early research stage, and several problems need to be solved in order for it to be used as a more stable and efficient therapeutic agent. The constant collection, maintenance, and sampling of reptile-derived anticancer components are important in the development of natural medicinal products. Library screening establishment, a systematic process for active substance identification, including sample purification and structural analysis, is essential for novel drug research. Additionally, the combination of reptile-derived anticancer components with various drug delivery system technologies such as medical implantable devices, small functional peptides, nanoparticles, liposomes, immunoliposomes, and antibody-drug conjugates (ADCs), might enable more safe and efficient pharmaceutical therapies [115,116,117,118,119,120,121]. If reptile-derived anticancer components and advanced technologies are actively fused and developed, an innovative cancer therapeutic drug platform could be organized based on a definite mechanism of action according to the individual components. In conclusion, reptile-derived components with therapeutic potential, such as extracts, peptides, sera, bile and venom, which are still in the early stages of research, will be an excellent novel source of medicines for cancer therapy in the future.

## Figures and Tables

**Table 1:** The anticancer effects of representative plant-derived natural products.

Natural Components	Source	Type of Cancer	Mechanism	Refs.
Vincristine	*Catharanthus roseus*	Acute lymphocytic leukemia Acute myeloid leukemia Hodgkin’s disease Neuroblastoma Lung cancer	Induction of apoptosis via binding to β-tubulin during cell division	[42]
Vinblastine	*Catharanthus roseus*	Leukemia Lymphoma	Induction of apoptosis via microtubule interference during cell division	[43]
Paclitaxel	*Taxus brevifolia*	Breast cancer Kaposi’s sarcoma Pancreatic cancer Gastric cancer	Inhibition of mitotic spindle assembly during cell division	[44]
Resveratrol	*Rheum rhaponticum*	Lymphoma Breast cancer	Suppression of Treg cells Inhibition of TGF-β production Interference interaction of PD-1/PD-L1	[45]
Curcumin	*Curcuma longa*	Breast cancer Lung cancer Gastric cancer Colon cancer	Induction of cell cycle arrest and apoptosis via inhibition of ERK, PI3K/Akt, Notch-1 and STAT-3	[46]
Capsaicin	*Capsicum annuum*	Osteosarcoma	Promotion of immunogenic cell death by mediating phagocytosis	[47]
Epigallocatechin-3-gallate (EGCG)	*Camelia sinensis*	Prostate cancer Melanoma	Induction of apoptosis and anti-angiogenesis	[48]
Parthenolide	*Tanacetum parthenium*	Breast cancer Lung cancer	Inhibition of JAK/STAT signaling Downregulation of EGFR expression	[49,50]
Ginsenoside Rg3	*Panax ginseng*	Breast cancer Colon cancer Gastric cancer Liver cancer	Induction of apoptosis via inhibition of ERK and Akt Inhibition of proliferation via G1 phase cell cycle arrest	[51]
Wogonin	*Scutellaria baicalensis*	Colon cancer Ovarian cancer	Inhibition of YAP1 expression Inhibition of VEGF, Bcl-2 and Akt signaling	[52,53]

**Table 2:** The anticancer effects of representative marine natural products.

Natural Components	Source	Type of Cancer	Mechanism	Refs.
Fucoidan	*Ascophyllum nodosum*	Colon cancer Breast cancer	Activation of macrophages and NK cells Induction of G1 phase cell cycle arrest	[54]
TZT-1027 (Soblidotin)	*Dolabella auricularia*	Lung cancer Colon cancer	Anti-angiogenesis Induction of apoptosis via microtubule interference during cell division	[55,56]
Heparin	*Dictyopteris delicatula*	Lung cancer Liver cancer Cervical cancer	Inhibition of PI3K/Akt signaling Anti-metastasis	[57,58,59]
Sansalvamide	*Fusarium solani*	Pancreatic cancer Colon cancer Prostate cancer Breast cancer	Induction of apoptosis via G1 phase cell cycle arrest	[60,61]
Plitidepsin	*Aplidium albicans*	Chronic lymphocytic leukemia	Inhibition of CXCL12 release from nurse-like cells (NLCs)	[62]
Dolastatin 10	*Dolabella auricularia*	Breast cancer Lung cancer Prostate cancer	Induction of apoptosis via microtubule interference during cell division	[63,64]
Halichondrin B (Eribulin)	*Halichondria okadai*	Breast cancer Liposarcoma	Induction of apoptosis via microtubule interference during cell division	[65]
Salinosporamide A (Marizomib)	*Salinispora tropica*	Lymphoma Breast cancer	Induction of apoptosis via inhibition of proteasome activity	[66,67,68,69]
C-nucleoside (Cytarabine)	*Cryptotheca crypta*	Leukemia	Inhibition of DNA synthesis	[70]
Jorumycin (Zalypsis)	*Jorunna funebris*	Leukemia Lung cancer Colon cancer	Induction of apoptosis via G1 phase cell cycle arrest	[68,71]

**Table 3:** The anticancer potentials of reptile-derived components.

Category	Natural Components	Source	Type of Cancer	Mechanism	Refs.
Extracts	Sulfated polysaccharide	*Gekko swinhonis*	Liver cancer	Inhibition of proliferation and differentiation	[72]
	Aqueous extracts	*Gekko swinhonis*	Liver cancer	Inhibition of growth Reduction in alpha fetoprotein	[73]
	Powder	*Gekko japonicus*	Esophageal carcinoma Sarcoma	Induction of apoptosis via decrease in VEGF and bFGF expression	[74]
	Ethanol extracts	*Cyrtopodion scabrum*	Breast cancer Colon cancer	Inhibition of growth and migration	[75]
	Aqueous extracts	*Eublepharis macularius*	Bladder cancer Cervical cancer Lung cancer	Induction of apoptosis via inhibition of PI3K/Akt signaling Induction of caspase-dependent apoptosis via G2/M phase cell cycle arrest	[76,77,78]
	Crocodile choline	*Crocodylus siamensis*	Gastric cancer	Induction of apoptosis via G2/M phase cell cycle arrest	[79]
	Methanol extracts	*Crocodylus palustris*	Prostate cancer	Induction of cell death	[80]
	Aqueous extracts of white blood cells	*Crocodylus siamensis*	Cervical cancer	Induction of mitochondria/caspase-3/caspase-9-mediated apoptosis Inhibition of proliferation, migration and invasion	[81,82]
	Aqueous extracts of white blood cells	*Crocodylus siamensis*	Lung cancer Prostate cancer Breast cancer Colorectal cancer	Induction of apoptosis via G2/M phase cell cycle arrest	[83]
	Aqueous extracts	*Chinemys reevesii*	Leukemia Liver cancer	Induction of cell death	[84]
	Aqueous extracts	*Cuora aurocapitata*	Leukemia Liver cancer	Induction of cell death	[84]
	Aqueous extracts	*Trachemys scripta*	Leukemia Liver cancer	Induction of cell death	[84]
Crude peptides	Sulfated polysaccharide–protein complex	*Gekko swinhonis*	Liver cancer	Inhibition of proliferation and migration	[85]
	Alcohol extracted crude peptides	*Gekko japonicus*	Liver cancer	Induction of apoptosis via Bcl-2/Bax pathway regulation Reduction in VEGF expression	[86]
	Polypeptide mixture	*Gekko japonicus*	Liver cancer	Induction of apoptosis Promotion of ROS-related processes and UPR	[87]
Sera	Blood serum	*Varanus salvator*	Cervical cancer Prostate cancer Breast cancer	Induction of cell death	[88]
	Blood serum	*Malayopython reticulatus*	Cervical cancer Prostate cancer Breast cancer	Induction of cell death	[88]
	Blood serum	*Cuora amboinensis karamoja*	Cervical cancer Prostate cancer Breast cancer	Induction of cell death	[88]
	Bile juice	*Crocodylus siamensis*	Lung cancer	Induction of mitochondria/caspase-3/caspase-9-mediated apoptosis	[89]
Venom	Lectin	*Macrovipera lebetina*	Breast cancer	Inhibition of integrin-mediated attachment and migration	[90]
	BnSP-6 Lys-49 PLA2	*Bothrops pauloensis*	Breast cancer	Induction of apoptosis Inhibition of adhesion, migration and angiogenesis	[91]
	NN-3	*Naja naja oxiana*	Breast cancer	Inhibition of proliferation	[92]
	Chlorotoxin	*Leiurus quinquestriatus*	Breast cancer	Inhibition of proliferation, migration and invasion	[93]
	Macrovipecetin	*Macrovipera lebetina*	Melanoma	Inhibition of proliferation, migration and invasion	[94]
	Crotoxin	*Crotalus durissus terrificus*	Breast cancer	Induction of apoptosis via G2/M phase cell cycle arrest Inhibition of ERK signaling	[95]
	Cytotoxin 2	*Naja naja oxiana*	Breast cancer Lung cancer	Induction of apoptosis via G1 phase cell cycle arrest Activation of caspase-3 and p38 signaling	[96,97]
	Daboialectin	*Daboia russelii*	Lung cancer	Induction of apoptosis Inhibition of migration	[98]
	Cytotoxin 1	*Naja atra*	Leukemia	Induction of necroptosis	[99]
	Disintegrin	*Echis multisquamatus*	Cervical cancer	Inhibition of proliferation	[100]
	Venom extracts	*Naja hage*	Liver cancer	Induction of cell death	[101]
	Venom extracts	*Vipera latifii*	Liver cancer	Induction of cell death	[102]
	Venom extracts	*Walterinnesia aegyptia*	Breast cancer	Induction of apoptosis Activation of caspase-3 pathway	[103]
	Recombinant protein cytotoxin 2	*Naja naja oxiana*	Melanoma	Induction of apoptosis via TGF-β-mediating SMAD signaling	[104]
	Jararhagin	*Bothrops jararaca*	Murine melanoma	Activation of caspase-3 pathway Suppression of tumor growth and metastasis	[105]
	L-Amino acid oxidase	*Cerastes vipera*	Breast cancer Liver cancer Lung cancer Prostate cancer Colon cancer	Induction of cell death	[106,107]
	Irradiated crude venom	*Cerastes cerastes*	Lung cancer Prostate cancer	Induction of apoptosis via G2/M phase cell cycle arrest	[108]

## Data Availability

Not applicable.
